# AlphaFold3-guided optimization of a photoactivatable endonuclease for top-down genome engineering

**DOI:** 10.1016/j.jbc.2025.110762

**Published:** 2025-09-24

**Authors:** Hideyuki Yone, Hiromitsu Kono, Moritoshi Sato, Kunihiro Ohta

**Affiliations:** 1 Department of Life Sciences, Graduate School of Arts and Sciences, The University of Tokyo, Tokyo, Japan; 2 Kanagawa Institute of Industrial Science and Technology (KISTEC), Kawasaki, Kanagawa, Japan; 3 Universal Biology Institute, The University of Tokyo, Tokyo, Japan; 4 Collaborative Research Institute for Innovative Microbiology, The University of Tokyo, Tokyo, Japan

**Keywords:** AlphaFold3, artificial intelligence, protein structure, protein engineering, optogenetics, endonuclease, restriction enzyme, DNA damage, DNA repair, genome rearrangement

## Abstract

Recent advances in protein structure prediction by artificial intelligence have enabled the rational design of engineered enzymes with enhanced activity and precise regulatory features. Here, we report the AlphaFold3-guided enhancement of MagMboI, a photoactivatable restriction enzyme designed for light-controlled top-down genome engineering. MagMboI is derived from the type II restriction enzyme MboI and functions through a split-protein strategy in which its N- and C-terminal fragments are fused to light-inducible dimerization modules. Upon exposure to blue light, these domains heterodimerize, restoring nuclease activity in a controlled manner. Using AlphaFold3, we modeled the structure of the MagMboI-DNA complex and gained structural insights into the interaction between MagMboI and its target DNA recognition sequence (5′-GATC-3′) required for Mg^2+^-dependent DNA cleavage. Comparing neighboring split-site variants, we identified an alternative split that increases the MagMboI–DNA interface area and enhances complex stability relative to the original construct. This redesigned variant (designated MagMboI-plus) preserves α-helical integrity while strengthening protein-DNA contacts. Although MagMboI-plus, when introduced in *Saccharomyces cerevisiae* cells, exhibited slightly increased DNA-cleavage activity *in vivo* upon blue light activation, it was found to induce more pronounced genomic rearrangements compared to the original MagMboI construct. These findings demonstrate that AlphaFold3-based prediction can accelerate functional improvements in engineered enzymes, providing a strategy for developing light-controlled genome engineering tools.

The ability to precisely predict structures of protein-substrate complexes has brought a major transition in modern molecular biology, offering a foundation for rational protein engineering and exploration of molecular-targeted medicine. For decades, researchers have diligently applied numerous computational methods to predict three-dimensional protein structures solely from amino acid sequences. The recent advent of deep learning has profoundly transformed the field, offering a promising solution to dramatically improve prediction accuracy (1).

The introduction of AlphaFold2 marked a major milestone, delivering near-experimental accuracy in monomeric protein structure prediction (2). Following this breakthrough, a diverse suite of deep learning-based prediction tools has been introduced: RoseTTAFold employs a three-track neural network to integrate sequence, distance, and coordinate information (3); ESMFold leverages protein language models to generate high-quality structures directly from single sequences without requiring multiple sequence alignments (4). AlphaFold3 now employs a generative diffusion approach to model complex biomolecular assemblies, including protein-protein interactions, protein-DNA complexes, and protein-ligand complexes (5). This advancement in computational biology enables the structure-guided design of multifunctional and engineered proteins for diverse biological applications.

Advances in protein structure prediction methods, such as AlphaFold3, now extend structure-informed design to biomolecules whose structures have not yet been experimentally determined, thereby opening new avenues for engineering proteins with externally controllable functions. Among these approaches, optogenetic enzymes have emerged as powerful tools for precise temporal and spatial control of cellular activity and genome manipulation (6). In particular, AlphaFold3 can predict protein-DNA and protein-ligand interactions at atomic resolution, providing a robust foundation for the rational design of light-dependent biological control mechanisms.

We previously established the MagTAQing system for light-controlled genome rearrangement by engineering the photoactivatable endonuclease MagMboI (7). In this system, the Type II restriction enzyme MboI, which recognizes and cleaves the palindromic sequence 5′-GATC-3′, was split into N- and C-terminal fragments. Each fragment was fused to a light-inducible Magnet dimerization domain, either nMag or pMag, which enables blue-light-dependent heterodimerization and reconstitution of nuclease activity in living cells (8, 9). Upon photoactivation, MagMboI induces DNA double-strand breaks (DSBs) at multiple genomic loci. These DSBs are repaired through DNA repair pathways such as homologous recombination or non-homologous end joining, often resulting in large-scale genome rearrangements, including translocations and loss-of-heterozygosity (LOH) events. However, the original MagMboI system did not fully match the extensive genome-rearrangement induction efficiency of the unsplit wild-type enzyme.

In this study, we employed AlphaFold3 to predict the structure of the MagMboI-DNA complex and elucidate the structural basis underlying its limited genome-rearrangement efficiency. The predicted structural model revealed how the enzyme recognizes its target DNA sequence (5′-GATC-3′) and coordinates Mg^2+^ ions at the catalytic center to mediate DNA cleavage. Analysis of neighboring split-site variants identified an alternative split pattern that increases the MagMboI-DNA interface area and enhances complex stability compared to the original construct. This redesigned variant, MagMboI-plus, was predicted to preserve α-helical integrity while strengthening protein-DNA contacts. MagMboI-plus exhibited slightly improved DNA cleavage activity and induced more extensive genome rearrangements compared to the original MagMboI construct. These results demonstrate that AlphaFold3-guided engineering can enhance the efficiency of light-controlled genome rearrangements.

## Results

### Prediction of the structure of heterodimeric MagMboI complex using AlphaFold3

**Figure 1:**
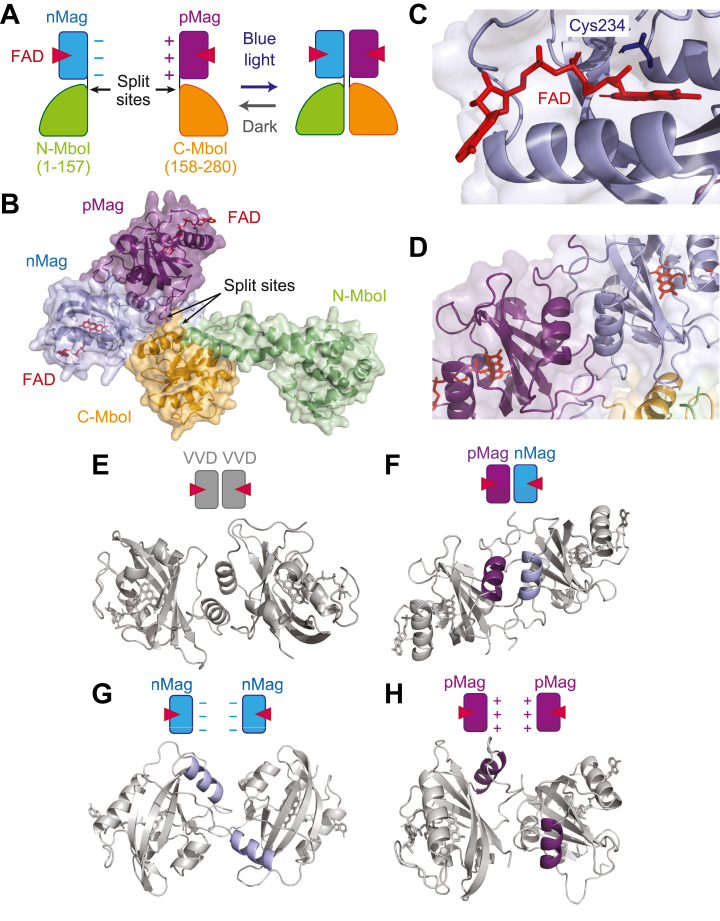
**Structural characterization of the heterodimeric MagMboI using AlphaFold3.***A*, schematic representation of photoactivatable restriction enzyme MagMboI. MboI (280 amino acids) is split at N157/C158 into N-MboI (residues 1–157) and C-MboI (residues 158–280), each fragment fused to a light-inducible heterodimerization Magnet domain (nMag or pMag) *via* peptide linkers. These Magnet domains are derived from the Vivid photoreceptor of *Neurospora crassa* and require flavin adenine dinucleotide (FAD) as a chromophore for dimerization. In the dark, the components remain dissociated and enzymatically inactive; exposure to *blue* light induces pMag-nMag heterodimerization, restoring MboI activity. The split sites (N157/C158) are indicated by *black arrows*. Color key: nMag (*blue*), pMag (*purple*), N-MboI (*green*), C-MboI (*orange*), peptide linker (*black*), FAD (*red*). *B*, AlphaFold3-predicted model of the MagMboI-FAD complex (average pLDDT = 83.4). *C*, close-up of the FAD-binding site in the Magnet domain, highlighting Cys234, which corresponds to Vivid Cys108 (the numbering offset results from N-terminal truncation of the Vivid-derived Magnet domain and the use of continuous residue numbering across the entire MagMboI fusion). This conserved cysteine forms a reversible C4a adduct with the FAD isoalloxazine ring upon blue-light exposure. *D*, close-up view of the pMag-nMag interface within the AlphaFold3-predicted MagMboI complex. *E–H*, comparative AlphaFold3 predictions of the specificity of dimerization domains. Dimerization domains are modeled in isolation from MboI to reveal their intrinsic pairing preferences. *E*, VVD-VVD homodimer (average pLDDT = 88.3). *F*, pMag-nMag heterodimer (average pLDDT = 92.3); *G*, nMag-nMag homodimer (average pLDDT = 88.0); *H*, pMag-pMag homodimer (average pLDDT = 86.7).

To elucidate the molecular mechanism of light-dependent activation of MagMboI, corresponding to MagMboI-8 described in Yone *et al.*, 2025 (7), we performed structural modeling using AlphaFold3. MagMboI consists of a split MboI endonuclease composed of N-MboI and C-MboI, which are fused to the blue light-inducible heterodimerization domains nMag and pMag, respectively (Fig. 1*A*). These domains are engineered from the *Neurospora crassa* photoreceptor Vivid (VVD) and require flavin adenine dinucleotide (FAD) as a chromophore to mediate light-responsive dimerization (8).

The predicted MagMboI-FAD complex (average pLDDT = 83.4) revealed that the two MboI fragments are brought into proximity upon pMag-nMag heterodimerization, forming a catalytically competent conformation (Fig. 1*B*). Consistent with this, structural alignment of AlphaFold3-predicted models confirmed that engineered MagMboI preserves the overall fold of wild-type MboI, supporting the integrity of its reconstituted conformation (Fig. S1). This spatial configuration suggests that blue light exposure restores enzymatic activity by reassembling the endonuclease structure. Inspection of the FAD-binding pocket within the Magnet domains revealed that conserved cysteine residues in nMag and pMag are positioned analogously to the photoreactive cysteine of wild-type Vivid (Fig. 1*C*). These residues are known to form a reversible covalent adduct with the isoalloxazine ring of FAD upon photoexcitation, facilitating conformational changes required for dimer formation (10). A close-up of the predicted pMag-nMag heterodimer interface demonstrated extensive intermolecular contacts that stabilize the dimerized state (Fig. 1*D*). The model successfully recapitulated electrostatic interactions, which have been linked to heterodimer selectivity in previous studies (8).

To evaluate dimerization specificity, we modeled four combinations of Magnet domains: VVD-VVD, pMag-nMag, nMag-nMag, and pMag-pMag (Fig. 1, *E*–*H*). The VVD-VVD pair formed a well-aligned homodimeric interface, consistent with its natural photodimerization function. In contrast, the nMag-nMag and pMag-pMag homodimers exhibited misaligned and poorly packed interfaces. The engineered pMag-nMag heterodimer displayed a compact, symmetrical interface with favorable structural features for light-dependent assembly.

Together, these structural predictions support a mechanism by which MagMboI activity is reconstituted upon blue light exposure through specific pMag-nMag heterodimerization. The orientation of catalytic fragments, conservation of the photoreactive cysteine, and selective interfacial interactions collectively enable tight control over light-activated restriction activity.

### Mechanistic insight into DNA recognition and cleavage by MagMboI

**Figure 2:**
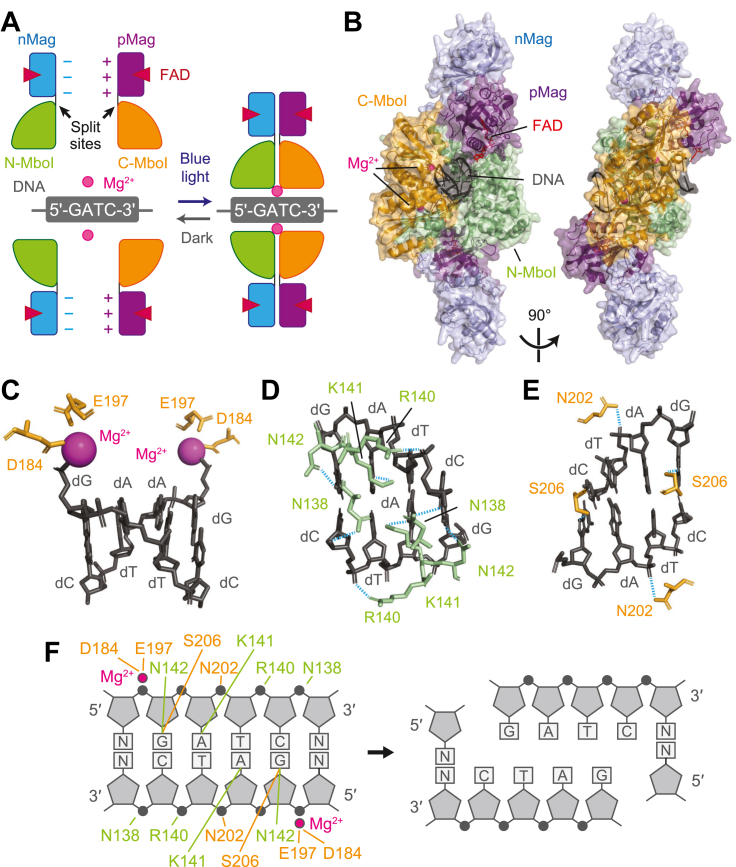
**Structural model of the MagMboI-DNA complex predicted by AlphaFold3.***A*, schematic representation of the MagMboI-DNA complex. MagMboI forms a light-inducible dimer and binds to the palindromic target sequence (5′-GATC-3′). DNA cleavage requires two MagMboI molecules, four FAD molecules, 2 Mg^2+^ ions, and a double-stranded DNA molecule containing the target site. The split sites are indicated by *black arrows*. Color key: nMag (*blue*), pMag (*purple*), N-MboI (*green*), C-MboI (*orange*), peptide linker (*black*), FAD (red), DNA (*gray*), Mg^2+^ ion (*magenta*). *B*, alphaFold3-predicted model of the MagMboI–DNA complex (average pLDDT = 87.1). Two MagMboI units are assembled around the DNA. *C*, Close-up view of the catalytic site showing Mg^2+^ ions coordinated by D184 and E197 of the C-MboI fragment. The ions are positioned adjacent to the phosphodiester bond 5′ to the guanine in the recognition sequence. *D and E*, detailed views of the hydrogen bonds formed by (*D*) N-MboI and (*E*) C-MboI with the recognition sequence. *F*, schematic summary of the predicted DNA recognition and cleavage mechanism. The positions of the 2 Mg^2+^ ions suggest that DNA cleavage occurs at the phosphodiester bond on the 5′ side of the guanine base in the recognition sequence, generating a four-base 5′ overhang (5′-GATC).

To explore the DNA-binding and cleavage properties of MagMboI, we performed structural modeling of the MagMboI-DNA complex using AlphaFold3. MboI is a Type II restriction enzyme that specifically recognizes and cleaves the sequence 5′-GATC-3′ in the form of a homodimer and requires Mg^2+^ ions as cofactors for DNA cleavage (11). Consequently, DNA cleavage by MagMboI requires two protein units, four FAD molecules, 2 Mg^2+^ ions, and a DNA duplex containing the 5′-GATC-3′ recognition site (Fig. 2*A*).

The predicted structure shows that two MagMboI molecules reassemble around the DNA to form a symmetric dimer (average pLDDT = 87.1) (Fig. 2*B*). Structural comparison with the wild-type MboI-DNA complex confirmed that MagMboI preserves a similar DNA-binding configuration, supporting the functional fidelity of the engineered enzyme (Fig. S2). A detailed analysis of the catalytic center showed that Mg^2+^ ions are coordinated by Asp184 and Glu197 in the C-MboI fragment (Fig. 2*C*). These residues correspond to Asp186 and Glu199 in wild-type MboI, which are reportedly essential for DNA binding and cleavage activity (12). The Mg^2+^ ions are located adjacent to the phosphodiester bond immediately 5′ to the guanine base in the recognition sequence, consistent with a catalytically competent geometry. Residue-level analysis identified potential hydrogen bonds involved in base-specific recognition (Fig. 2, *D* and *E*). Ser206 of C-MboI was positioned to form a hydrogen bond with the guanine base within the 5′-GATC-3′ recognition sequence, while Lys141 of N-MboI was predicted to interact with the adenine base at the same recognition sequence, providing structural support for sequence specificity.

A schematic summary of the predicted cleavage mechanism is shown in Figure 2*F*. Based on the position of the Mg^2+^ ions and their proximity to the scissile bond, the model predicts that cleavage occurs immediately upstream of the guanine base, generating a four-base 5′ overhang (5′-GATC). This predicted cleavage pattern is consistent with the biochemical activity of wild-type MboI, supporting the functional fidelity of the engineered MagMboI complex.

To further examine whether this modeling approach could be applied to other restriction enzymes, we predicted the DNA-bound structures of BspHI (5′-TCATGA-3′) and SmaI (5′-CCCGGG-3′), for which no experimental DNA-bound structures have been reported. As shown in Figure S3, the predicted locations of Mg^2+^ ions in both complexes were consistent with the biochemically characterized cleavage sites, indicating that this method can accurately infer the catalytic geometry of other restriction endonucleases beyond MagMboI.

### Structure-guided improvement in DNA cleavage activity of MagMboI

**Figure 3:**
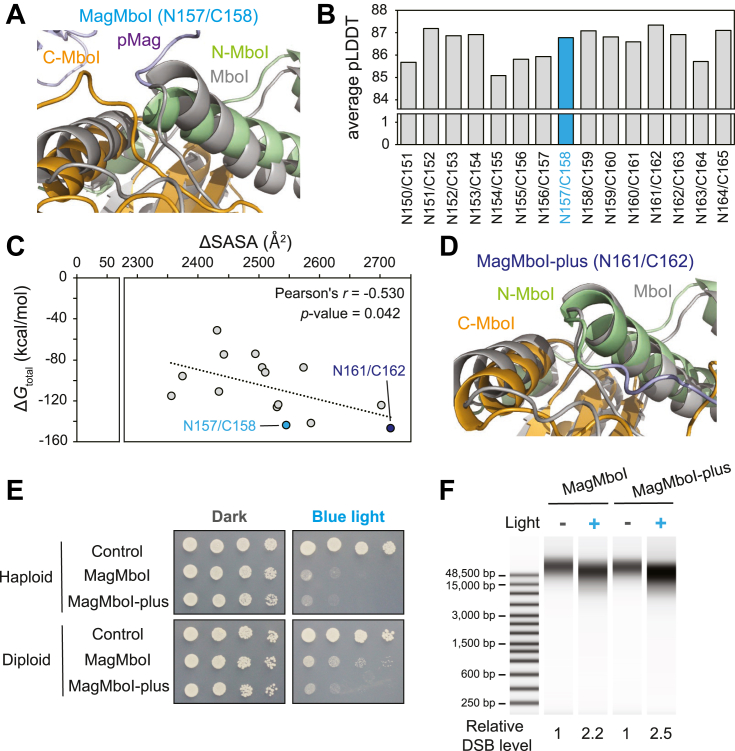
**Structure-guided engineering of the Magnet split site enhances MagMboI activity.***A*, structural overlay of MagMboI and intact MboI. The original split site for Magnet insertion (N157/C158) is located within an α-helix that is critical for DNA recognition. *B*, average per-residue pLDDT scores predicted by AlphaFold3 for each variant. *C*, scatter plot of ΔSASA (Å^2^) *versus* the free energy change Δ*G* (kcal/mol) of the entire MagMboI–DNA complex for each variant, showing a significant inverse correlation (Pearson's *r* = −0.53, *p* = 0.042). *D*, AlphaFold3-based models of a repositioned split site at N161/C162 (termed MagMboI-plus). *E*, spotting assay comparing dark *versus* blue-light conditions in yeast expressing MagMboI-plus or the original MagMboI. *F*, blue-light-dependent DNA cleavage was assessed by quantifying total DNA fragmentation in MagMboI-activated cells using the TapeStation system.

To improve light-inducible DNA cleavage by MagMboI, we first reconsidered the original split site (N157/C158), located at the terminus of the DNA-recognition α-helix and likely to perturb its helical structure (Fig. 3*A*). Next, we generated AlphaFold3 models for a series of neighboring candidate split sites and calculated average pLDDT scores, which were relatively high and uniform across these variants, indicating comparable model confidence (Fig. 3*B*). We calculated ΔSASA (difference in solvent-accessible surface area) by subtracting the SASA of the individual protein and DNA from that of the complex. We also estimate the stability of the entire MagMboI–DNA complex for each variant by determining the total Gibbs free energy change (Δ*G*) of the complex. Analysis of the relationship between ΔSASA and Δ*G* revealed a significant inverse correlation (Pearson's *r* = −0.53, *p* = 0.042), indicating that larger interface areas correspond to enhanced complex stability (Fig. 3*C*). Evaluation of neighboring positions identified a split at N161/C162 that outperformed the original construct in both ΔSASA and Δ*G*. In the corresponding AlphaFold3 model, this split site is located on the solvent-exposed face of the α-helix, preserving backbone continuity while strengthening protein-DNA contacts (Fig. 3*D*). We designated this engineered construct “MagMboI-plus” (Fig. S4).

We next investigated whether the modified construct exhibited enhanced light-inducible enzymatic activity by introducing MagMboI-plus into *Saccharomyces cerevisiae* and assessing growth under blue light conditions. In haploid cells, MagMboI-plus and the original MagMboI construct resulted in comparable levels of light-dependent growth inhibition. However, in diploid cells that are known to exhibit stronger resistance to DNA breaks, MagMboI-plus expression led to a more severe growth defect than the original MagMboI upon illumination, suggesting that MagMboI-plus has improved activity in response to blue light (Fig. 3*E*).

To assess DNA cleavage activity, we conducted a digestion assay of genomic DNA by the TapeStation system (Agilent Technologies). MagMboI-plus produced slightly increased DNA fragments compared to the original MagMboI construct (10.8% increase in DNA fragmentation, relative DSB level = 2.455 *versus* 2.215), indicating that the split site repositioning modestly enhances light-inducible nuclease activity (Fig. 3*F*). Collectively, these findings indicate that structure-guided repositioning of the Magnet split site can enhance MagMboI function, underscoring the potential of AlphaFold3 for the rational design of allosterically controlled restriction enzymes.

### Enhanced induction of genome rearrangements by MagMboI-plus

To assess whether the increased enzymatic activity of MagMboI-plus enhances genome rearrangement efficiency, we employed the MagTAQing system previously established for light-inducible genome engineering. In this system, the diploid *S. cerevisiae* strain SYW4, which was generated by fusing haploid SK1 and S288C strains with 0.7% single-nucleotide polymorphisms (SNPs), was transformed with a plasmid expressing MagMboI-plus. The SNPs enable us to identify the sites of chromosomal rearrangements after whole-genome sequencing (7, 13). After blue light exposure, the photoactivated cells were plated, and individual colonies were isolated. From these, 23 isolates exhibiting altered cellular morphology were obtained as previously described (7).

**Figure 4:**
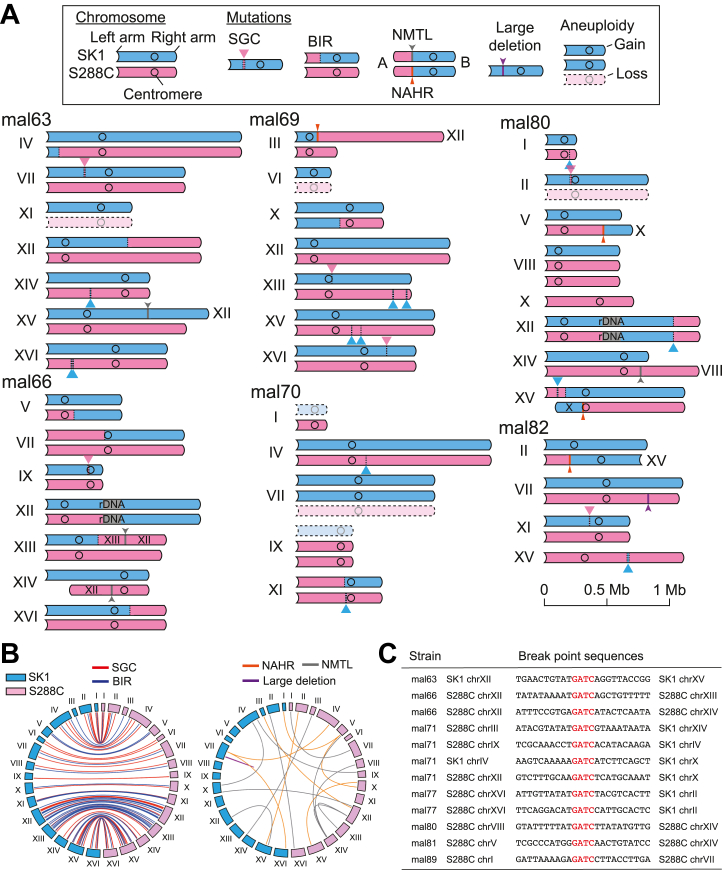
**Large-scale genome rearrangements induced by MagMboI-plus.***A*, schematic representation of rearranged chromosomes in representative MagMboI-plus-activated (MagTAQed) isolates. Various classes of rearrangement events, including short gene conversion (SGC), break-induced replication (BIR), non-homologous end joining-mediated translocation (NMTL), and non-allelic homologous recombination (NAHR), are mapped onto a reference karyotype. *B*, Circos plot depicting genome-wide structural variations in MagTAQed isolates. Each arc represents a chromosomal rearrangement and is color-coded by rearrangement class. *C*, breakpoint sequences of NMTL events. All cleavage sites exactly match the MboI recognition sequence (5′-GATC-3′).

Whole-genome sequencing of the 23 isolates revealed a diverse spectrum of structural variations similar to those observed in previous studies (7, 13): 88 loss-of-heterozygosity (LOH) events, 19 translocations (TLs), 41 instances of aneuploidy, one large deletion, and 10 small-scale mutations (Fig. S5). LOH tracts were further classified into short gene conversion (SGC) and break-induced replication (BIR) (Figs. 4, *A* and *B*, S6). Translocations were categorized into non-homologous end joining-mediated translocations (NMTLs) and non-allelic homologous recombination (NAHR) events occurring at repetitive elements such as Ty retrotransposons. Analysis of NMTL breakpoint sequences revealed perfect matches to the canonical MboI recognition site (5′-GATC-3′), confirming that MagMboI-plus maintains strict sequence specificity (Fig. 4*C*).

**Figure 5:**
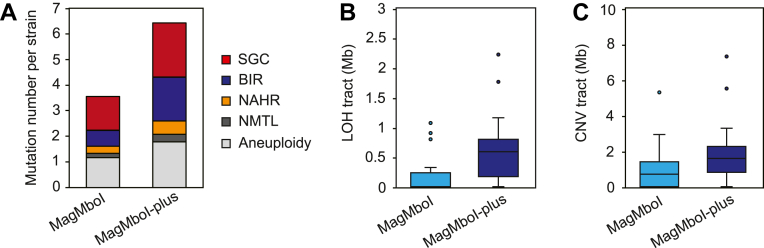
**Extensive genome rearrangements induced by MagMboI-plus.***A*, number of structural variants per isolate, including SGC, BIR, NAHR, NMTL, and aneuploidy events. *B*, Total length of loss-of-heterozygosity (LOH) tracts per isolate (*p* < 0.001). *C*, Total length of copy number variation (CNV) tracts per isolate (*p* < 0.05). *p* values were calculated using a two-sided Mann-Whitney-Wilcoxon test. For all box plots, center lines represent medians, box limits denote interquartile ranges (IQR), and whiskers extend to 1.5 × IQR. Sample sizes: MagMboI, *n* = 18; MagMboI-plus, *n* = 23.

The frequency of genome rearrangements (6.4 rearrangement events per isolate) was significantly higher (1.77-fold) in strains expressing MagMboI-plus than in those expressing the original MagMboI construct (3.6 rearrangement events per isolate), with increased frequencies of SGC, BIR, and aneuploidy events (Fig. 5*A*). In particular, BIR events occurred about 2.8 times more frequently. Furthermore, MagMboI-plus isolates exhibited significantly greater sum of lengths of both LOH and CNV tracts, indicating more extensive chromosomal rearrangements compared to the original MagMboI construct (Fig. 5, *B* and *C*). Together, these findings demonstrate that MagMboI-plus enhances both the efficiency and scope of light-induced genome rearrangements, further expanding the applicability of the MagTAQing system.

## Discussion

Recent breakthroughs in computational protein design and precise structural modeling now allow us to conduct rational protein engineering. In this study, we investigated the restriction enzyme MagMboI, whose DNA cleavage activity can be controlled by blue-light irradiation, by using AlphaFold3 to predict its 3D structure in the presence of Mg^2+^, FAD, and substrate DNA, aiming to optimize its split site. For several split patterns, we estimated the difference in solvent-accessible surface area (ΔSASA) with and without substrate DNA and calculated the overall change in total Gibbs free energy (Δ*G*). Since we observed a negative correlation between ΔSASA and Δ*G*, among the various split patterns, the one with the largest ΔSASA and the smallest Δ*G* was considered to make strong and stable contact with DNA while causing minimal impact on the overall structure; this construct was designated as “MagMboI-plus”. MagMboI-plus indeed exhibited stronger genome DNA cleavage activity in cells under blue light irradiation compared to the original MagMboI and led to an increased frequency of DNA rearrangements. On the other hand, the breakpoints of translocations caused by NMTLs retained the MboI recognition sequence, similar to the original MagMboI, confirming that sequence specificity was preserved. This simple optimization method using AlphaFold3 is expected to facilitate the optimization of split patterns for various photoactivatable enzymes and the improvement of DNA-cleaving enzymes.

Previous studies have also proposed computational simulation-based methods for optimizing light- or chemical-controllable proteins. One prominent example is SPELL, which employs a “split energy” scoring function to identify split sites that avoid structurally critical regions and enable effective inducible reassembly. Using SPELL, researchers have designed split kinases, GTPase regulators, proteases, and other proteins with low spontaneous reassembly and robust ligand- or light-induced activation. (14, 15). Chemically inducible trimerization (CIT) demonstrates how split FKBP and FRB can be rationally partitioned to form a rapamycin-inducible ternary complex, enabling recruitment of a third component and expanding the versatility of synthetic signaling systems (16). Additionally, SPORT employs Rosetta-based calculations of interfacial energetic changes (ΔΔ*G*) and ΔSASA to identify key buried residues for mutagenesis, enabling the design of sparse mutant libraries that yield split proteins with tightly regulated conditional reconstitution (17). Finally, integration of molecular dynamics and QM/MM simulations enabled the identification of unexpected mutation sites that promote efficient light-induced β-strand dissociation in split GFP, while maintaining structural stability under dark conditions (18). Taken together, these complementary computational and experimental strategies are rapidly expanding the design space for the next generation optogenetic and chemogenetic protein tools.

AlphaFold2 achieved near-experimental accuracy in predicting monomeric protein structures, fundamentally transforming rational protein engineering (2). Following this breakthrough, AlphaFold3 now delivers atomic-level models of fully heterogeneous biomolecular assemblies. Its unified, diffusion-based architecture predicts protein–protein, protein–DNA, protein–RNA, and protein–ligand complexes (5).

Complementing these developments, RoseTTAFold and its derivatives provide rapid, end-to-end modeling of both protein-nucleic acid interactions and more general biomolecular systems. In particular, RoseTTAFoldNA extends the three-track network of RoseTTAFold to predict protein-RNA and protein-DNA complexes with confidence estimates comparable to state-of-the-art docking approaches (19), while RoseTTAFold All-Atom further generalizes the graph-based representation of small molecules, metals and covalent modifications alongside proteins and nucleic acids, enabling accurate, unified modeling of assemblies containing arbitrary non-polymeric components (20). These complementary tools are useful for estimating whether proteins artificially split to control activity can form appropriate 3D structures and establish more effective interactions with their substrates upon activation without any information of co-crystal structures. While these methods differ in terms of computational load and availability as cloud-based services, it is important to choose the appropriate tool depending on the specific purpose.

The TAQing system was originally developed to induce large-scale genome rearrangements by transient heat activation of the restriction enzyme TaqI, generating DNA double-strand breaks (DSBs) at multiple sites and yielding diverse phenotypic variants in *S. cerevisiae* and *Arabidopsis thaliana* (13). The Ex-TAQing approach substituted TaqI with other nucleases such as MseI, whose frequent recognition sequences allowed for rapid and high-efficiency genome reorganization across diploid and polyploid plants (21). To avoid transgenesis, TAQing2.0 employs cell-penetrating peptides to introduce purified restriction enzymes tagged with a nuclear localization signal directly into the cell nucleus, eliminating both gene transfer steps and foreign DNA incorporation and yielding non-genetically modified organisms (22, 23).

The MagTAQing system integrated photoactivatable MagMboI into the TAQing framework, permitting blue-light-controlled DSB induction with spatiotemporal precision. With the MagMboI variant, we observed about only 3.6 rearrangements per isolate, whereas the intact TaqI-based TAQing system yielded about 5.1 events (7, 13). On the other hand, the MagMboI-plus variant induced about 6.4 rearrangement events per isolate, exceeding the performance of the original MagTAQing and even the TaqI-based TAQing protocol (1.77-fold and 1.25-fold, respectively). The increase in genomic rearrangements caused by MagMboI-plus, compared to the original MagMboI, was considerably more pronounced than the modest (∼10%) rise in intracellular DNA cleavage frequency. Although the exact reason remains unclear, one possible explanation is that MagMboI-plus may be more stable in the cell or recognize and cleave target sequences more precisely *in vivo* than the original MagMboI. To further clarify, the rearrangement frequencies reported here are based on isolates selected according to abnormal cellular morphology. While this approach efficiently enriches for rearranged genomes, the estimates should be regarded as approximate values. A more comprehensive evaluation will require whole-genome sequencing of randomly chosen isolates, independent of morphological appearance, in future studies. Excessive DSB induction can be counterproductive by provoking cell death and suppressing proliferation, which can prevent recovery of desired isolates, strains, or cell lines in some contexts. Accordingly, the construct and effective activity should be tuned to the application by selecting an appropriate variant with appropriate nuclease strength and adjusting illumination parameters (*e.g.*, duration and pulse frequency).

AlphaFold3-guided engineering of MagMboI has elevated light-controlled genome rearrangements to match or surpass the classic TaqI-based method, establishing MagTAQing as a versatile platform for synthetic genome engineering in a wide range of organisms. Blue-light-induced genome rearrangement affords precise spatiotemporal control and is well suited for applications in tissue-specific consequences of induced DSBs in animal models like other optogenetic tools. Targeted genome rearrangements can be leveraged to characterize tissue-specific rearrangement patterns and to assess genome instability across distinct disease models, enabling more precise construction and evaluation of pathophysiological models.

## Experimental procedures

### AlphaFold3

The amino acid sequences of proteins and DNA sequences were submitted to the AlphaFold3 online server (https://alphafoldserver.com/) to generate predicted complex structures. For protein modeling, the nuclear localization signal (NLS) and N-terminal His tag were removed from the MagMboI construct. The Magnet domains are N-terminal truncated versions of the photoreceptor Vivid (VVD, residues 37–186) containing electrostatic mutations (I52R/M55R in pMag and I52D/M55G in nMag) that enable light-dependent heterodimerization (8). The resulting models were visualized and analyzed using PyMOL (v3.0). Hydrogen bonds between MagMboI and DNA were identified using a donor-acceptor distance threshold of ≤ 3.5 Å. ΔSASA (Å^2^), defined as the buried surface area upon MagMboI–DNA complex formation, was quantified in UCSF ChimeraX (v1.9) by subtracting the solvent-accessible surface areas of the isolated protein and DNA from that of the assembled complex. The total Gibbs free energy change (Δ*G*_total_) of the MagMboI-DNA complex was calculated using the “Stability” command in FoldX (v5.1) (24). Average pLDDT (predicted Local-Distance Difference Test) was obtained by averaging per-residue pLDDT scores across all residues, yielding a single composite measure of model confidence.

### Yeast strains and culture

The *S. cerevisiae* strain SYW4 (*MATa/a ura3-52/ura3 lys2-801/lys2 ho::LYS2 leu2Δ/LEU2 ade2-101/ADE2 trp1-Δ63/TRP1 his3-Δ200/HIS3 arg4-bgl/ARG4 cyh2-z/CYH2 flo8-1/flo8:hyg*) was generated by protoplast fusion between two haploid strains: S799 (derived from the SK1 background, *flo8Δ*) and YPH499 (derived from the S288 C background) in a previous study (7).

Cells were grown at 30 °C in either yeast extract-peptone-dextrose (YPD; 10 g/L yeast extract, 20 g/L peptone, 20 g/L glucose) or synthetic defined/monosodium glutamic acid medium lacking leucine (SD/MSG-Leu; 1.7 g/L yeast nitrogen base without amino acids, 0.69 g/L CSM-Leu, 1.0 g/L monosodium ʟ-glutamate, and 20 g/L glucose). Plasmid DNA was transformed into yeast cells using the canonical LiAc/SS carrier DNA/PEG method (25). Transformants were selected on SD/MSG-Leu plates and cultured at 30 °C. Strains used in this study were listed in Table S1.

### Plasmid construction

The MagMboI constructs encode a split version of the restriction enzyme MboI from *Moraxella bovis*, codon-optimized for expression in *S. cerevisiae*. The enzyme is divided into N-terminal (N-MboI) and C-terminal (C-MboI) fragments, each fused to light-inducible dimerization modules, nMag and pMag, respectively. MagMboI contains a Magnet split site between residues N157 and C158, as reported in our previous study (7). In contrast, MagMboI-plus has an alternative split site introduced between residues N161 and C162. Both constructs are expressed under the control of the copper-inducible *CUP1* promoter. Each split protein includes an N-terminal nuclear localization signal (NLS), and the two units are co-expressed from a single transcript separated by a self-cleaving P2A peptide. The final constructs were cloned into the pRS415 vector. Plasmid sequences were verified by Sanger sequencing.

### Spot assay

Haploid (YPH499) and diploid (SYW4) *S. cerevisiae* strains harboring either an empty vector (pRS415) or MagMboI constructs were cultured at 30 °C in SD/MSG-Leu medium. Ten-fold serial dilutions of each culture were spotted onto SD/MSG-Leu agar plates and incubated at 30 °C for 48 h in either darkness or under continuous blue light (465–475 nm, 2–3 mW/cm^2^) using an LED-based illumination system (NSPB510AS, Nichia; 4972822310628, Daiso Industries Co., Ltd).

### MagTAQing

The diploid strain SYW4 harboring the pRS415-MagMboI-plus construct was grown overnight at 30 °C in SD/MSG-Leu medium under dark conditions to prevent premature light activation. Cultures were then diluted into fresh SD/MSG-Leu and incubated for 4 h in the presence of 150 μM CuSO_4_ to induce MagMboI-plus expression. During the exponential phase, cells were harvested, washed with sterile distilled water, and exposed to blue light (470 nm) for 30 min to activate the restriction enzyme. After light exposure, cells were spread onto YPD agar plates and incubated at 30 °C in the dark for several days until visible colonies emerged. Out of over 1000 colonies evaluated, 23 isolates displaying altered cellular morphology were identified and designated as MagTAQed isolates.

### Quantification of cleaved DNA

The diploid strain SYW4 carrying MagMboI constructs was cultured overnight in SD/MSG-Leu medium at 30 °C under dark conditions. The overnight culture was diluted into fresh SD/MSG-Leu medium and incubated until the exponential phase. Cells were then exposed to blue light (470 nm) to trigger MagMboI activation. Following illumination, cells were fixed with 1% formaldehyde for 10 min at room temperature, followed by the extraction of genomic DNA. DNA fragmentation was assessed using the Agilent 4200 TapeStation system (Agilent Technologies) with Genomic DNA ScreenTape. Sample processing and data acquisition followed the protocols provided by the manufacturer. The amount of cleaved DNA was quantified using TapeStation Analysis Software, with fragmented DNA defined as fragments ranging in size from 200 to 48,500 base pairs. The relative DSB level was calculated by normalizing the amount of fragmented DNA to that obtained under dark conditions, serving as the baseline for each construct.

### Whole-genome sequencing analysis

Whole genome sequencing analysis of MagTAQed isolates was performed as described in previous studies (7, 26). First, genomic DNA was isolated using the Dr GenTLE High Recovery kit for yeast (Takara Bio Inc). DNA was sheared to ∼300 bp using a Covaris M220 ultrasonicator (Covaris, LLC). Sequencing libraries were prepared with the NEBNext Ultra II DNA Library Prep Kit and NEBNext Multiplex Oligos for Illumina (New England Biolabs) and paired-end reads were generated on an Illumina HiSeq X 10 platform.

Sequence reads were aligned to the SYW4 diploid reference genome and to the parental haploid genomes (SK1 and S288C) using BWA-MEM (27), based on the pipeline described in Yone *et al.*, 2025 (7). Variant calling for SNVs and indels was carried out with GATK (28). LOH events were identified based on read depth and allele frequency. Long-range LOH tracts extending to chromosome ends were classified as BIR, and interstitial LOH regions were assigned as SGC.

Structural variations such as NHEJ-mediated translocations (NMTLs) and non-allelic homologous recombination (NAHR) events were inferred from chimeric and discordant read signatures using SAMtools (29) and validated by PCR (Table S2). Genome rearrangements were visualized using OMGenomics Circa 1.2.2 and copy number alterations including aneuploidy were assessed *via* coverage and allele ratio using IGV (30). Mutations observed in control strain were removed from the final analysis. Total LOH tract length was calculated as the cumulative length of homozygous regions resulting from SGC or BIR. Similarly, total CNV tract length was defined as the sum of chromosomal regions exhibiting altered DNA dosage caused by structural variations such as NAHR, NMTLs, and aneuploidy. The isolate mal61, which exhibited haploidization following MagMboI induction, was excluded from the analysis due to the complete loss of heterozygosity. Mutations of MagTAQed isolates were listed in Table S3.

### Statistical analysis

Statistical comparisons for Figure 5, *B* and *C* were performed using a two-sided Mann-Whitney-Wilcoxon test in R. For Figure 5*B*, total lengths of LOH tracts per isolate were compared between MagMboI (*n* = 18) and MagMboI-plus (*n* = 23) isolates, yielding a *p* < 0.001. For Figure 5*C*, total lengths of CNV tracts per isolate were similarly compared, with a *p* < 0.05. Box plots indicate the median (center line), interquartile range (box limits), and 1.5 × IQR (whiskers).

## Data availability

Sequencing data generated in this study have been deposited in the DDBJ/EMBL/GenBank database under the BioProject accession number PRJDB35671. The assembled genome sequences of the parental strains YPH499 and S799 are available under BioProject accession number PRJDB5253. All additional data supporting the findings of this study are available from the corresponding authors upon reasonable request.

## Supporting information

This article contains Supporting information.

## Conflict of interest

The authors declare that they do not have any conflicts of interest with the content of this article.
