# Supplementary material for: AlphaFold3-guided optimization of a photoactivatable endonuclease for top-down genome engineering
Source: J Biol Chem. 2025 Sep 24;301(11):110762. doi: 10.1016/j.jbc.2025.110762 (PMC12569821; doi:10.1016/j.jbc.2025.110762)
Supplement: Supplementary Figures [file mmc1.pdf]

Supporting information

AlphaFold3-guided optimization of a photoactivatable endonuclease for top-down genome engineering

Hideyuki Yone[1], Hiromitsu Kono[1], Moritoshi Sato[1,2], and Kunihiro Ohta[1,3,*]

[1]Department of Life Sciences, Graduate School of Arts and Sciences, The University of Tokyo, Komaba 3-8-1, Meguro-ku, Tokyo 153-8902, Japan

[2]Kanagawa Institute of Industrial Science and Technology (KISTEC), 3-2-1 Sakado, Takatsu-ku, Kawasaki, Kanagawa 213-0012, Japan.

[3]Universal Biology Institute, The University of Tokyo, Hongo 7-3-1, Bunkyo-Ku, Tokyo 113-0033, Japan.

*Corresponding author: K. Ohta (kohta-pub2@bio.c.u-tokyo.ac.jp)

Tel & Fax, +81-3-5465-8834

Description of supporting information

Supporting figures

Figure S1. Structural alignment of predicted models of MagMboI and MboI.

Figure S2. Structural alignment of predicted MagMboI–DNA and MboI–DNA complexes.

Figure S3. AlphaFold3 modeling of restriction enzymes other than MboI.

Figure S4. Constructs of MagMboI-plus.

Figure S5. List of mutations detected in 23 isolates

Figure S6. Genome rearrangements of MagTAQed isolates.

Supporting tables

Table S1. Strains used in this study

Table S2. Primer sets used to confirm translocations

Table S3. Mutations in MagTAQed isolates

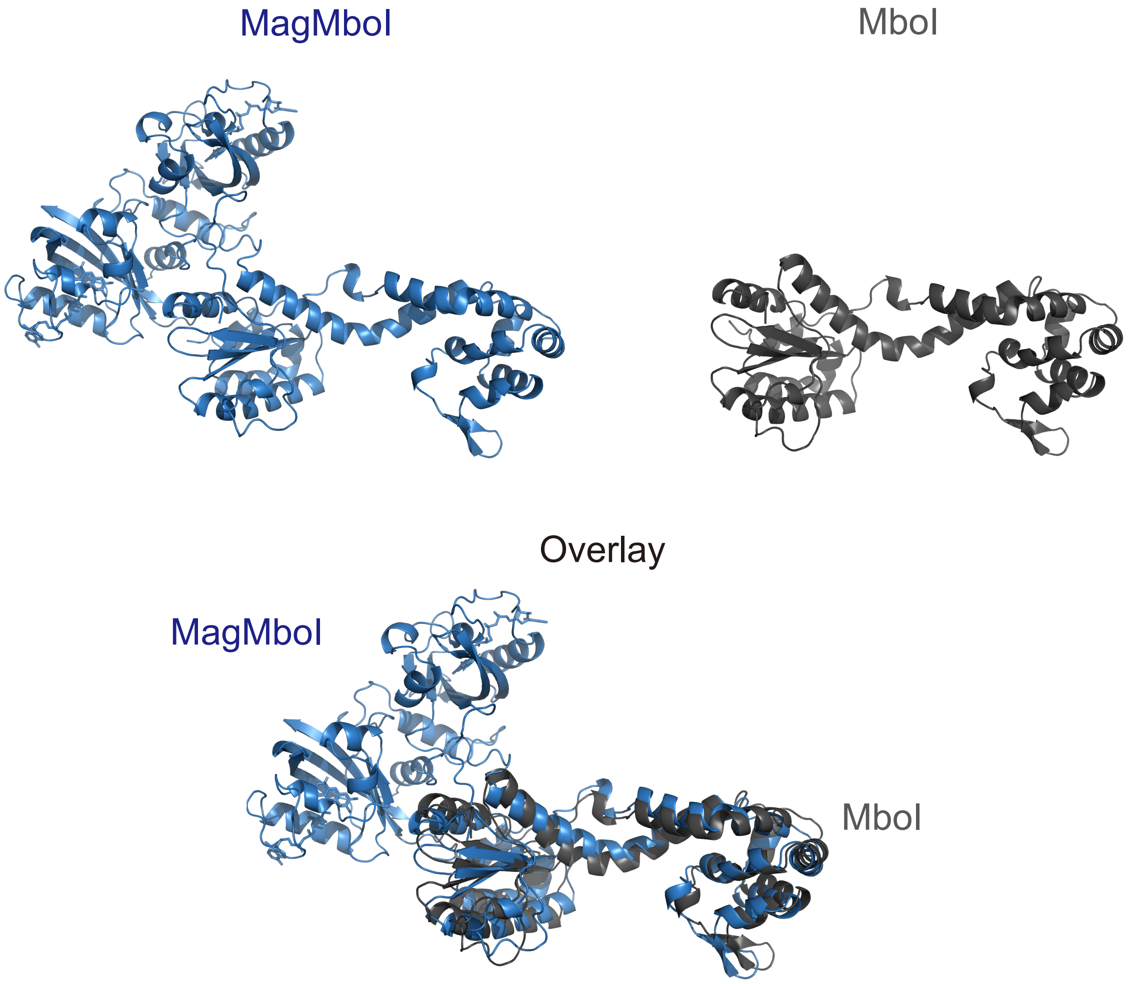

Figure S1. Structural alignment of predicted models of MagMboI and MboI. Overlay of structural models predicted by AlphaFold3 reveals shared folding architecture between engineered MagMboI and wild-type MboI.

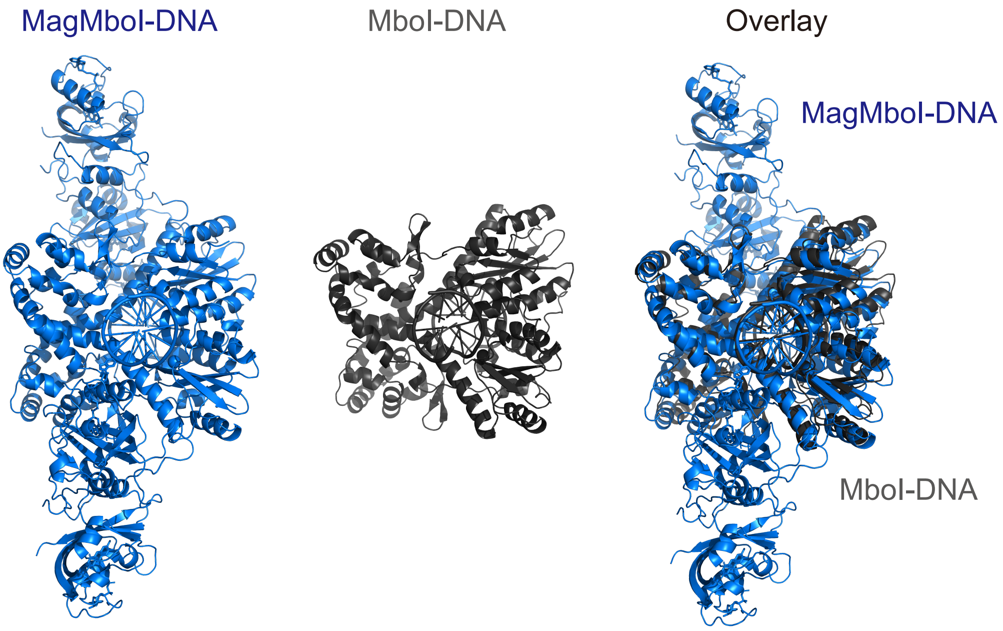

Figure S2. Structural alignment of predicted MagMboI–DNA and MboI–DNA complexes.

Overlay of AlphaFold3-predicted DNA-bound structures reveals conserved DNA-binding architecture between engineered MagMboI and wild-type MboI.

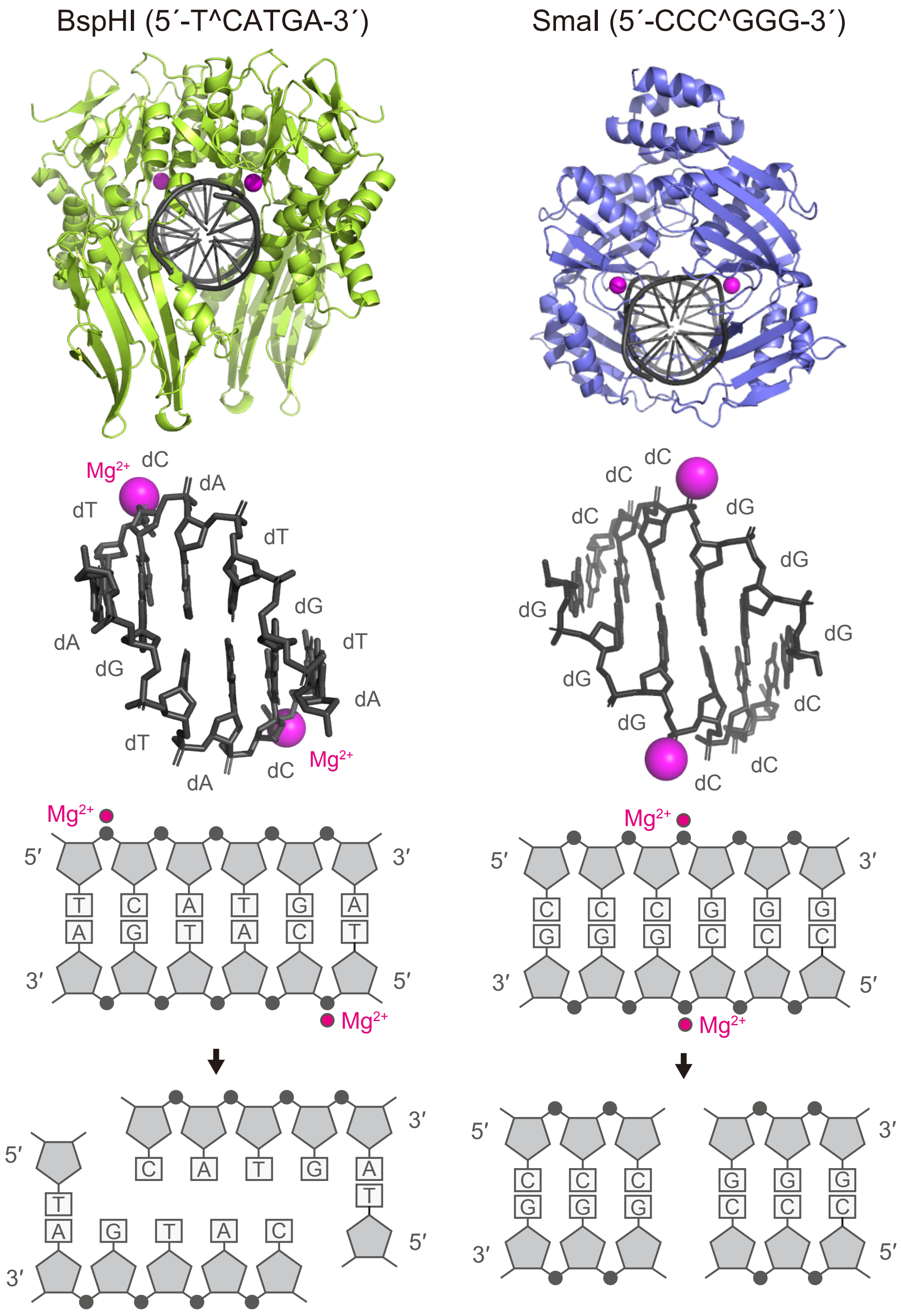

Figure S3. AlphaFold3 modeling of restriction enzymes other than MboI.
AlphaFold3-predicted structures of DNA-bound BspHI and SmaI reveal conserved recognition and cleavage geometries consistent with their biochemically characterized cleavage sites.

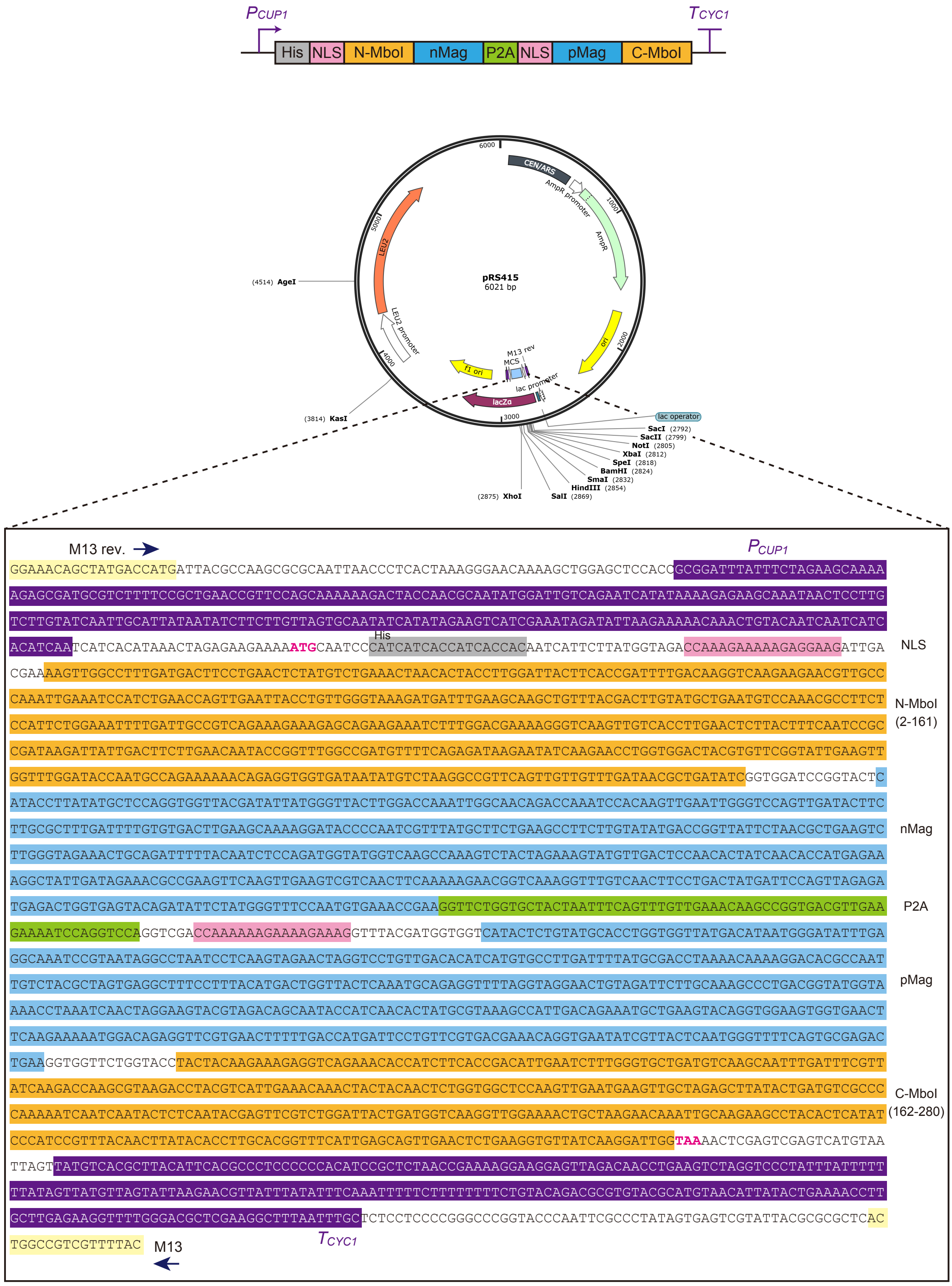

Figure S4. Constructs of MagMboI-plus.

Schematic diagrams of MagMboI-plus constructs used in this study.

Type		Numbers of events
Loss-of-heterozygosity (LOH)	Short gene conversion (SGC)	49
	Break-induced replication (BIR)	39
Translocation (TL)	NHEJ-mediated translocation (NMTL)	12
	Non-allelic homologous recombination (NAHR)	7
Aneuploidy	Gain	17
	Loss	24
Structural variation	Large-scale deletion	1
Small-scale mutation	Single nucleotide variant (SNV)	7
	Insertion (INS)	1
	Deletion (DEL)	2

Figure S5. List of mutations detected in 23 isolates.

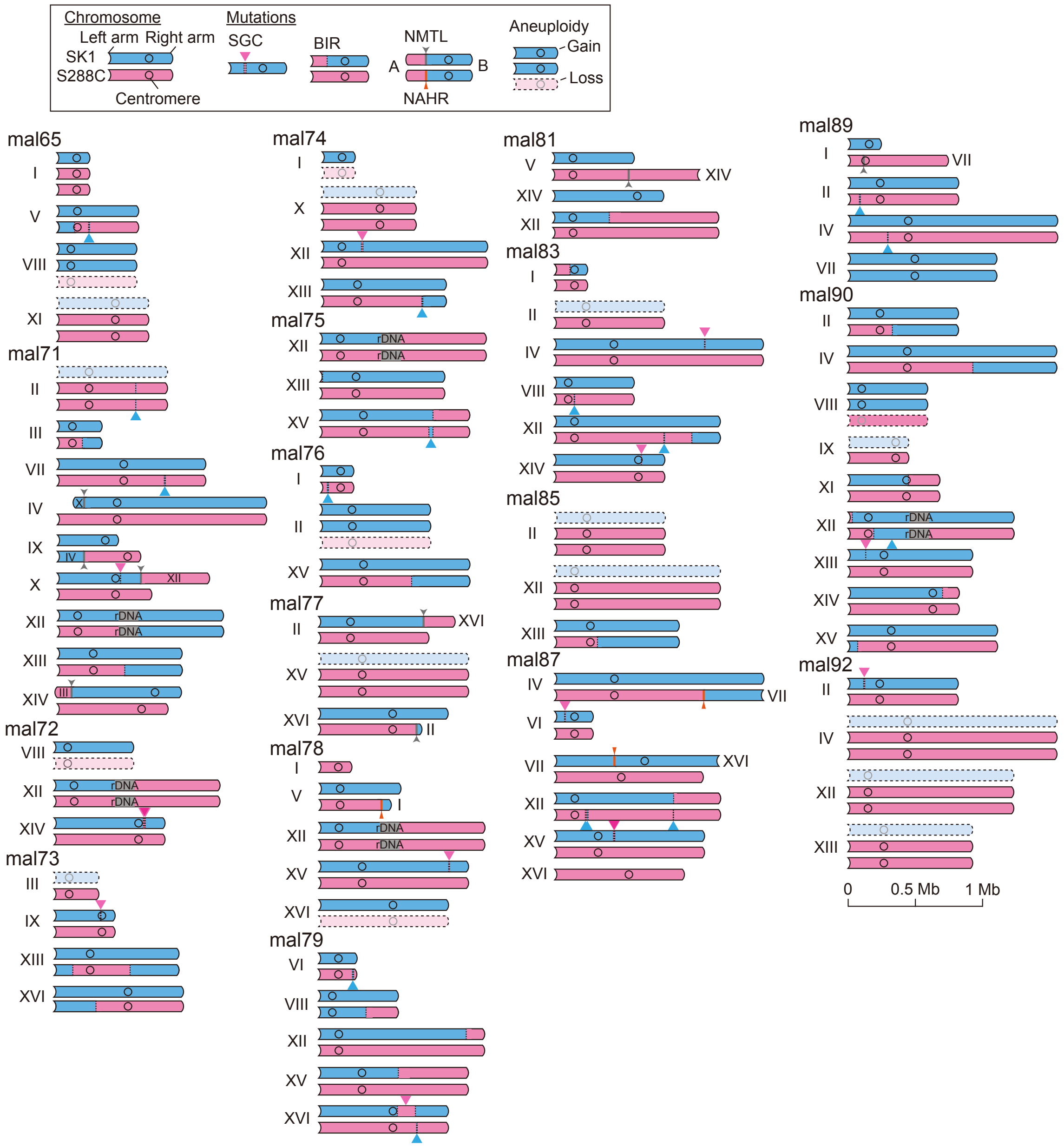

Figure S6. Genome rearrangements of MagTAQed isolates.

Schematic representation of chromosomal rearrangements in MagTAQed isolates other than those shown in Figure 4A.
